# Rezvilutamide plus docetaxel in chemotherapy‐naive metastatic castration‐resistant prostate cancer patients after progression on abiraterone: A multi‐centre, open‐label, phase II trial

**DOI:** 10.1002/ctm2.70649

**Published:** 2026-04-16

**Authors:** Zhenhua Liu, Bo Chen, Tao Dai, Shusuan Jiang, Hong Luo, Hong Liao, Dexin Yu, Zhiwen Chen, Dahong Zhang, Zeng Li, Zhiqiang Zhang, Hong Bai, Feng Liu, Jiaqin Lin, Yike Wang, Ping Feng, Qiang Wei

**Affiliations:** ^1^ Department of Urology Institute of Urology West China Hospital Sichuan University Chengdu China; ^2^ Department of Urology Surgery Hunan Cancer Hospital Changsha China; ^3^ Department of Uro‐Oncology Chongqing University Cancer Hospital Chongqing China; ^4^ Department of Urological Oncology Sichuan Cancer Hospital Chengdu China; ^5^ Department of Urology Second Affiliated Hospital of Anhui Medical University Hefei China; ^6^ Department of Urology The First Affiliated Hospital of the Chinese People's Liberation Army Medical University Chongqing China; ^7^ Department of Urology Zhejiang Provincial People's Hospital Hangzhou China; ^8^ Clinical Development‐Oncology Jiangsu Hengrui Pharmaceuticals Co., Ltd Shanghai China; ^9^ Clinical Statistics Jiangsu Hengrui Pharmaceuticals Co., Ltd Shanghai China; ^10^ Clinical Pharmacology Jiangsu Hengrui Pharmaceuticals Co., Ltd Shanghai China; ^11^ Clinical Trial Centre and National Medical Products Administration Key Laboratory for Clinical Research and Evaluation of Innovative Drugs West China Hospital Sichuan University Chengdu China

**Keywords:** androgen receptor antagonist, docetaxel, metastatic castration‐resistant prostate cancer

## Abstract

**Background:**

A multi‐centre, 2‐part, phase II study assessed adding rezvilutamide (a novel androgen receptor antagonist) to docetaxel in abiraterone‐refractory, metastatic castration‐resistant prostate cancer (mCRPC) patients who were naive to chemotherapy. Herein, we report results from part 1.

**Methods:**

Part 1 encompassed both dose‐escalation and dose‐expansion phases. Eligible patients received oral rezvilutamide (160/240 mg, once daily [QD]) plus intravenous docetaxel (75 mg/m^2^, on the first day of every 3‐week cycle) for more than 10 cycles, followed by maintenance with rezvilutamide monotherapy (240 mg, QD). Rezvilutamide was administered daily starting from the second day of cycle 1. Docetaxel was co‐administered with oral prednisone at 5 mg twice daily. Safety served as the primary objective in Part 1.

**Results:**

From 2, December 2020, to 5, August 2021, 36 patients were eligible for enrolment (18 patients with rezvilutamide 160 mg plus docetaxel and 18 patients with rezvilutamide 240 mg plus docetaxel). No one experienced dose‐limiting toxicity. 32 (88.9%) patients experienced grade 3 or higher treatment‐related adverse events. At week 12, 67.7% of the 31 evaluable patients showed a response in prostate‐specific antigen (PSA) levels. Among all 36 patients, the median times were 10.5 months (95% CI: 5.6–14.1) for PSA progression, 13.8 months (95% CI: 8.4–19.2) for radiological progression‐free survival and 16.2 months (95% CI: 12.9–22.5) for overall survival. The limitations were a small sample size and a non‐randomised design.

**Conclusions:**

Rezvilutamide plus docetaxel followed by maintenance with rezvilutamide monotherapy demonstrated good tolerability and promising efficacy in chemotherapy‐naive, abiraterone‐refractory, mCRPC patients.

## Graphical abstract

• Rezvilutamide plus docetaxel followed by maintenance with rezvilutamide monotherapy was well tolerated in chemotherapy‐naive metastatic castration‐resistant prostate cancer (mCRPC) patients who have progressed after abiraterone.

• Rezvilutamide plus docetaxel followed by maintenance with rezvilutamide monotherapy demonstrated promising efficacy in mCRPC patients who have progressed after abiraterone.

• These results provide a new treatment option for chemotherapy‐naive, abiraterone‐refractory, mCRPC patients.

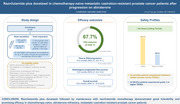

## 1 INTRODUCTION

In 2022, an estimated 1.5 million men were newly diagnosed with prostate cancer worldwide, and about 397 000 died from it, underscoring its status as the second most prevalent cancer and fifth leading cause of cancer death in men globally.^1^ The increasing incidence and burden of prostate cancer present significant treatment challenges.^2^, ^3^ Given that prostate cancer relies on androgens for growth, anti‐androgen therapy has been the backbone therapy for prostate cancer.^4^, ^5^ However, most patients treated with anti‐androgen therapies eventually develop resistance, leading to metastatic castration‐resistant prostate cancer (mCRPC).^6^

Abiraterone (an inhibitor of CYP17A1), enzalutamide [a second‐generation androgen receptor (AR) antagonist] or docetaxel are the standard first‐line therapy for mCRPC.^7^, ^8^ A phase I/II study showed abiraterone followed by enzalutamide benefits mCRPC patients.^9^ Thus, it is anticipated that a subset of mCRPC patients would receive abiraterone as a first‐line treatment. Docetaxel is advised for chemotherapy‐naive mCRPC patients who have received novel hormone therapy.^7^, ^8^ However, docetaxel shows limited effectiveness in those who have progressed following abiraterone treatment.^10^, ^11^, ^12^, ^13^, ^14^, ^15^ There remains an unmet need for this population.

Inhibition of the AR pathway boosts prostate cancer cells’ sensitivity to docetaxel and reverses resistance.^16^, ^17^ In addition to inhibiting mitosis in prostate cancer cells, docetaxel also inhibits AR pathway activation, curbing non‐mitotic tumour cell growth.^18^, ^19^ The aforementioned preclinical studies show AR antagonists and docetaxel synergise in prostate cancer. A phase Ib trial of enzalutamide combined with docetaxel in mCRPC patients, including those who had failed abiraterone, showed that all five abiraterone‐resistant patients had a PSA response (decline ≥50%).^20^ Exploration of novel AR antagonists plus docetaxel for chemotherapy‐naive mCRPC patients who had failed abiraterone is promising.

Rezvilutamide (formerly SHR3680) is an orally active second‐generation AR antagonist.^21^, ^22^, ^23^ Rezvilutamide demonstrated promising anti‐tumour activity and safety in a phase I/II mCRPC clinical research.^21^ Herein, we initiated a phase II study that was conducted to assess rezvilutamide plus docetaxel followed by maintenance with rezvilutamide monotherapy in chemotherapy‐naive mCRPC patients who had progressed after abiraterone.

## 2 PATIENTS AND METHODS

### 2.1 Study design and participants

This study is a multi‐centre, open‐label, 2‐part, phase II trial conducted at 7 centres (ClinicalTrials.gov identifier: NCT04603833). Part 1 employed a two‐phase design, beginning with dose escalation and then proceeding to dose expansion. Part 2 was a randomised, controlled, open‐label, multi‐centre clinical trial. Here, we reported the findings of part 1.

Eligible patients were 18 years or elder with prostate cancer without neuroendocrine differentiation or small‐cell histology, had progressed on abiraterone, maintained a castrate testosterone level (≤50 ng/dL or 1.73 nmol/L), exhibited metastases in bone or soft‐tissue, had an Eastern Cooperative Oncology Group performance status of 0–1, a life expectancy of 3 months or more and sufficient organ function. Disease progression was characterised by any of the following: PSA progression, indicated by at least two rising PSA levels separated by a minimum of one week with the final result being ≥2 ng/mL; soft tissue progression as per RECIST version 1.1; or bone disease progression according to PCWG3 criteria. Key exclusion criteria encompassed previous treatment with novel AR antagonists and prior cytotoxic chemotherapy for mCRPC.

The ethics committee of each study centre approved the study protocol and protocol amendments. The Declaration of Helsinki and the Good Clinical Practice guidelines were adhered to. All participants formally consented in writing before any study‐related procedures.

### 2.2 Procedures

The dose escalation phase utilised a standard 3+3 design. Patients were administered rezvilutamide [planned escalating doses, 160 and 240 mg; orally (PO), once daily (QD)] plus docetaxel (75 mg/m^2^, intravenously on the first day of every 3‐week cycle) for ≤10 cycles, followed by maintenance with rezvilutamide monotherapy (240 mg QD). Rezvilutamide was administered daily starting from the second day of cycle 1. Docetaxel was co‐administered with oral prednisone at 5 mg twice daily. A phase I/II rezvilutamide study (40–480 mg QD) in mCRPC patients established 240 mg QD as the phase III recommended dose without reaching maximum tolerated dose.^21^ Therefore, the study selected 160 mg QD and 240 mg QD for dose escalation, with 240 mg QD as the maintenance dose for rezvilutamide monotherapy in this study. Dose‐limiting toxicities (DLTs) were observed during Cycle 1.

In the dose‐expansion phase, 1 or 2 dose groups would be expanded based on the results of the dose escalation phase. The study treatment persisted until the earliest disease progression, death, unacceptable toxicity, or withdrawal by patient or investigator. Details on treatment interruptions, dose reductions of rezvilutamide and docetaxel and prednisone adjustments are provided in the Supporting Information Material.

### 2.3 Outcomes

Safety served as the primary objective. The secondary endpoints encompassed PK parameters and efficacy endpoints. PK parameters included peak concentration (C_max_), area under the time‐concentration curve (AUC) from time 0 to 24 h (AUC_0–24 h_), time to C_max_ (T_max_), ratio of AUC_0–24 h_ in cycle 2 versus AUC_0‐24 h_ in cycle 1 (cycle 2/cycle 1 ratio of AUC_0–24 h)_, ratio of C_max_ in cycle 2 versus C_max_ in cycle 1 (cycle 2/cycle 1 ratio of C_max)_. Efficacy endpoints included the rate of PSA response at the 12th week, incidence of PSA decline ≥50% from baseline during the treatment, objective response rate (ORR), disease control rate (DCR), time to PSA progression, radiological progression‐free survival (PFS) and overall survival (OS). The definition of efficacy endpoints is available in the Supporting Information Material.

### 2.4 Assessments

For the definition of DLTs, see Supporting Information Material. AEs, laboratory tests, vital signs, physical examination, electrocardiograms and echocardiography were monitored for safety assessments. Adverse events were documented from informed consent until 30 days post‐final dose or the initiation of a new antitumor treatment, whichever came first. All AEs were graded using the National Cancer Institute Common Terminology Criteria for Adverse Events version 5.0.

PSA concentrations were measured at baseline, every 3 weeks from week 4 during the combined treatment period, and every 6 weeks during the rezvilutamide monotherapy period. Tumour radiographic imaging assessments (CT or MRI following RECIST version 1.1, or bone scan [^99m^Tc] following the PCWG3 criteria) were conducted at baseline, week 13, and every 12 weeks thereafter, until disease progression or initiation of new anti‐tumour therapy. Complete response (CR) or partial response (PR) per RECIST version 1.1 was required to be confirmed ≥4 weeks after the first documented response. Progressive disease identified through a bone scan required confirmation after a minimum of 6 weeks. Survival was evaluated bi‐monthly throughout the follow‐up period. Details regarding the blood sample collection for the PK analysis are in the Supporting Information Material.

### 2.5 Statistical analysis

There was no formal statistical consideration for sample size calculation. In the dose escalation phase, each dose group comprised either three or six patients. In the dose expansion phase, a total of approximately 30 patients would be enrolled.

DLTs were assessed in all patients from the dose‐escalation phase who either completed the 21‐day evaluation period or encountered any DLT within the period. Safety and efficacy (except for PSA response at week 12, ORR and DCR) were determined for the treated population (defined as those receiving one or more doses). Data for PSA response at the 12th week were derived from patients who received at least one dose of the study drug and provided one or more PSA measurements at or beyond week 12. ORR and DCR were analysed in patients who were administered one or more doses of study treatment with documented measurable baseline target lesions. PK concentration and PK parameter analyses were conducted on patients who were administered one or more doses of the study treatment and had one or more qualified PK concentration and PK parameter results.

Baseline demographics and safety were summarised using descriptive statistics. The point estimates for PSA response at week 12, PSA decline ≥50% from baseline, ORR and DCR were provided with two‐sided 95% CIs calculated via the Clopper‐Pearson method. Time‐to‐event outcomes, such as time to PSA progression, radiological progression‐free survival and overall survival, were assessed using the Kaplan–Meier method with two‐sided 95% CIs calculated via the Brookmeyer–Crowley method. PK parameters were evaluated using non‐compartmental analysis in Phoenix WinNonlin (version 8.1 or higher), while additional statistical analyses were performed using SAS (version 9.4 or higher).

## 3 RESULTS

### 3.1 Patients

From 2 December 2020 to 5 August 2021, 36 patients participated, each receiving at least one dose of the study drug (18 patients received rezvilutamide 160 mg combined with docetaxel, and 18 patients received rezvilutamide 240 mg combined with docetaxel; Figure S1). All patients discontinued study treatment permanently. Ten (27.8%) patients were given subsequent systemic antitumor therapy after discontinuing the study treatment (Table S1). By the data cutoff (29 August 2024), the median follow‐up duration was 16.1 months (range: 2.5–36.6). Table 1 provides a summary of the patients’ demographics and baseline characteristics.

**TABLE 1:** Demographic and baseline characteristics.

	Rezvilutamide 160 mg plus docetaxel (*n* = 18)	Rezvilutamide 240 mg plus docetaxel (*n* = 18)	Overall (n = 36)
Age, years	68.5 (49.0–78.0)	72.0 (58.0–79.0)	69.5 (49.0–79.0)
ECOG performance status			
0	3 (16.7)	7 (38.9)	10 (27.8)
1	15 (83.3)	11 (61.1)	26 (72.2)
Gleason score			
˂8	1 (5.6)	5 (27.8)	6 (16.7)
≥8	16 (88.9)	13 (72.2)	29 (83.3)
Missing	1 (5.6)	0	1 (2.8)
PSA, ng/mL	81.7 (2.7–5993.0)	144.0 (2.9–3720.0)	115.1 (2.7–5993.0)
Testosterone, ng/dL	2.6 (2.5–17.3)	2.5 (0–17.0)	2.5 (0–17.3)
Alkaline phosphatase, U/L	186.5 (84.0–599.0)	126.0 (70.8–456.0)	147.7 (70.8–599.0)
Lactate dehydrogenase, U/L	227.0 (153.0–1077.0)	211.9 (128.9–1297.2)	223.3 (128.9–1297.2)
Bone metastases			
≤10	5 (27.8)	10 (55.6)	15 (41.7)
>10	13 (72.2)	8 (44.4)	21 (58.3)
Prior treatment			
Surgery	5 (27.8)	3 (16.7)	8 (22.2)
Radiotherapy	0	3 (16.7)	3 (8.3)
Systemic therapy	18 (100.0)	18 (100.0)	36 (100.0)
Abiraterone	18 (100.0)	18 (100.0)	36 (100.0)
Duration of prior treatment with abiraterone	10.9 (1.9–42.7)	10.8 (1.3–31.1)	10.9 (1.3–42.7)
Prior cytotoxic chemotherapy for mHSPC	2 (11.1)	1 (5.6)	3 (8.3)

*Note*: Data are *n* (%) or median (range).

Abbreviations: ECOG, Eastern Cooperative Oncology Group; mHSPC, metastatic hormone‐sensitive prostate cancer; PSA, prostate‐specific antigen.

During the dose escalation phase, six patients (three received rezvilutamide 160 mg combined with docetaxel, and three received rezvilutamide 240 mg combined with docetaxel) were enrolled and received at least one dose of study treatment. No one experienced DLTs. Subsequently, both groups were expanded to include 18 patients each.

### 3.2 Safety

The safety assessment included all 36 patients. Thirty‐six (100.0%) patients experienced TRAEs, with the most common being decreased neutrophil count [33 (91.7%)] and decreased white blood cell count [33 (91.7%)], followed by anaemia [31 (86.1%); Table 2 and S1]. Grade 3 or higher TRAEs were observed in 32 (88.9%) patients. The most frequently grade 3 or higher TRAEs were decreased neutrophil count [27 (75.0%)], decreased white blood cell count [24 (66.7%)] and anaemia [10 (27.8%)].

**TABLE 2:** Treatment‐related adverse events.

	Rezvilutamide 160 mg plus docetaxel (*n* = 18)		Rezvilutamide 240 mg plus docetaxel (*n* = 18)		Overall (n = 36)	
	Any grade	Grade 3–5	Any grade	Grade 3–5	Any grade	Grade 3–5
Any	18 (100.0)	14 (77.8)	18 (100.0)	18 (100.0)	36 (100.0)	32 (88.9)
Neutrophil count decreased	15 (83.3)	11 (61.1)	18 (100.0)	16 (88.9)	33 (91.7)	27 (75.0)
White blood cell count decreased	15 (83.3)	7 (38.9)	18 (100.0)	17 (94.4)	33 (91.7)	24 (66.7)
Anaemia	14 (77.8)	5 (27.8)	17 (94.4)	5 (27.8)	31 (86.1)	10 (27.8)
Fatigue	12 (66.7)	0 (0)	10 (55.6)	3 (16.7)	22 (61.1)	3 (8.3)
Alopecia	8 (44.4)	0 (0)	12 (66.7)	0 (0)	20 (55.6)	0 (0)
Decreased appetite	7 (38.9)	1 (5.6)	10 (55.6)	0 (0)	17 (47.2)	1 (2.8)
Lymphocyte count decreased	5 (27.8)	1 (5.6)	10 (55.6)	2 (11.1)	15 (41.7)	3 (8.3)
Hypertriglyceridaemia	8 (44.4)	0 (0)	5 (27.8)	0 (0)	13 (36.1)	0 (0)
Hypercholesterolaemia	6 (33.3)	0 (0)	3 (16.7)	0 (0)	9 (25.0)	0 (0)
Hypoalbuminaemia	1 (5.6)	0 (0)	7 (38.9)	0 (0)	8 (22.2)	0 (0)
Edema peripheral	3 (16.7)	0 (0)	4 (22.2)	0 (0)	7 (19.4)	0 (0)
Platelet count decreased	2 (11.1)	0 (0)	5 (27.8)	0 (0)	7 (19.4)	0 (0)
Hypoesthesia	2 (11.1)	0 (0)	4 (22.2)	0 (0)	6 (16.7)	0 (0)
Nausea	1 (5.6)	0 (0)	5 (27.8)	0 (0)	6 (16.7)	0 (0)
Neurotoxicity	4 (22.2)	0 (0)	2 (11.1)	0 (0)	6 (16.7)	0 (0)
Pruritus	2 (11.1)	0 (0)	4 (22.2)	0 (0)	6 (16.7)	0 (0)
Constipation	1 (5.6)	0 (0)	4 (22.2)	0 (0)	5 (13.9)	0 (0)
Hyperglycaemia	3 (16.7)	0 (0)	2 (11.1)	0 (0)	5 (13.9)	0 (0)
Sleep disorder	3 (16.7)	0 (0)	2 (11.1)	0 (0)	5 (13.9)	0 (0)
Weight decreased	1 (5.6)	0 (0)	4 (22.2)	0 (0)	5 (13.9)	0 (0)
Aspartate aminotransferase increased	1 (5.6)	0 (0)	3 (16.7)	0 (0)	4 (11.1)	0 (0)
Diarrhoea	0	0 (0)	4 (22.2)	0 (0)	4 (11.1)	0 (0)
Hot flush	3 (16.7)	0 (0)	1 (5.6)	0 (0)	4 (11.1)	0 (0)
Hyperlipidaemia	3 (16.7)	0 (0)	1 (5.6)	0 (0)	4 (11.1)	0 (0)
Pneumonia	1 (5.6)	1 (5.6)	3 (16.7)	2 (11.1)	4 (11.1)	3 (8.3)
Peripheral swelling	1 (5.6)	0 (0)	3 (16.7)	2 (11.1)	4 (11.1)	2 (5.6)
Rash	3 (16.7)	0 (0)	1 (5.6)	0 (0)	4 (11.1)	0 (0)
Vomiting	1 (5.6)	0 (0)	3 (16.7)	0 (0)	4 (11.1)	0 (0)
Blood alkaline phosphatase increased	0	0 (0)	3 (16.7)	1 (5.6)	3 (8.3)	1 (2.8)
Chest discomfort	0	0 (0)	3 (16.7)	1 (5.6)	3 (8.3)	1 (2.8)
Febrile neutropenia	0	0 (0)	3 (16.7)	2 (11.1)	3 (8.3)	2 (5.6)
Hypocalcaemia	0	0 (0)	3 (16.7)	2 (11.1)	3 (8.3)	2 (5.6)
Hypokalaemia	0	0 (0)	3 (16.7)	1 (5.6)	3 (8.3)	1 (2.8)
Urinary tract infection	1 (5.6)	1 (5.6)	2 (11.1)	0 (0)	3 (8.3)	1 (2.8)
Proteinuria	0	0 (0)	2 (11.1)	1 (5.6)	2 (5.6)	1 (2.8)
Bacteraemia	0	0 (0)	1 (5.6)	1 (5.6)	1 (2.8)	1 (2.8)
Blood infection	1 (5.6)	1 (5.6)	0	0 (0)	1 (2.8)	1 (2.8)
Cardiac failure	0	0 (0)	1 (5.6)	1 (5.6)	1 (2.8)	1 (2.8)
Coronary artery disease	1 (5.6)	1 (5.6)	0	0 (0)	1 (2.8)	1 (2.8)
Hematuria	0	0 (0)	1 (5.6)	1 (5.56)	1 (2.8)	1 (2.8)
Hypertension	0	0 (0)	1 (5.6)	1 (5.56)	1 (2.8)	1 (2.8)
Interstitial lung disease	0	0 (0)	1 (5.6)	1 (5.56)	1 (2.8)	1 (2.8)
Neutropenia	1 (5.6)	1 (5.6)	0	0 (0)	1 (2.8)	1 (2.8)
Sepsis	1 (5.6)	1 (5.6)	0	0 (0)	1 (2.8)	1 (2.8)

*Note*: Data are *n* (%). Treatment‐related adverse events of any grade occurring in at least 10% of total patients and all treatment‐related adverse events of grade 3–5 in total patients are reported.

Serious TRAEs were reported in 12 (33.3%) patients, with pneumonia [3 (8.3%)], anaemia [2 (5.6%)] and decreased appetite [2 (5.6%)] being the most common ones (Tables S2 and S3). TRAEs leading to dose reductions or interruptions occurred in 13 patients (36.1%), with 7 patients (19.4%) affected by rezvilutamide‐related AEs and 9 patients (25.0%) affected by docetaxel‐related AEs (Table S2). Six (16.7%) patients discontinued treatment due to TRAEs, all of which were associated with docetaxel, with none linked to rezvilutamide (Table S2). Three (8.3%) patients died due to AEs (1 with respiratory‐circulatory failure, and 2 with tumour progression), and no deaths were considered treatment‐related (Tables S2 and S4).

### 3.3 PK

The PK concentration and parameter analyses of docetaxel, when combined with rezvilutamide, were evaluated in 36 and 34 patients, respectively. Figure S2 illustrates the plasma concentration‐time profiles of docetaxel for both groups. Detailed PK parameters of docetaxel are listed in Table S5. The geometric mean cycle 2/cycle 1 ratios of AUC_0–24 h_ and C_max_ for docetaxel were .735 and .781 with 160 mg of rezvilutamide, and.591 and.680 with 240 mg of rezvilutamide.

### 3.4 Efficacy

**FIGURE 1:**
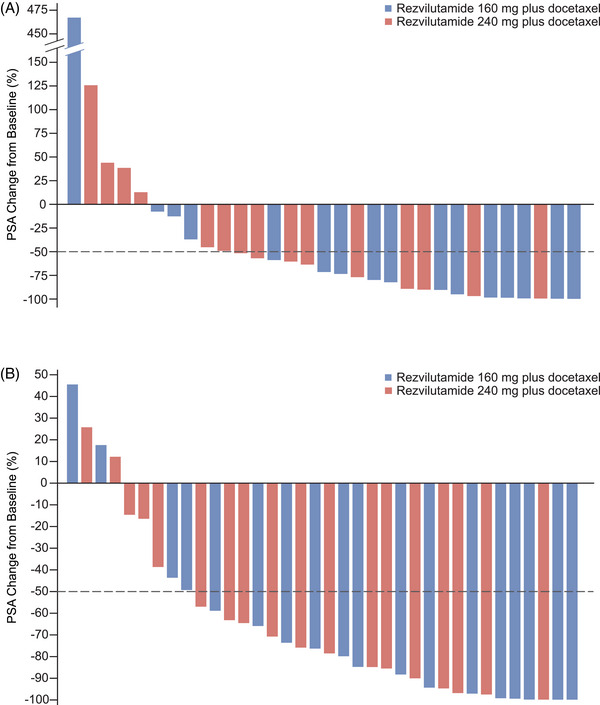
Percentage change in PSA from baseline. (A) Decrease of PSA at week 12 from baseline. (B) Maximum decrease of PSA from baseline. PSA, prostate‐specific antigen.

By week 12, 21 (67.7%; 95% CI, 48.6–83.3) of the 31 evaluable patients who received at least one dose of the study treatment and had at least one PSA level measurement at week 12 or later achieved a PSA response (Table 3). At week 12, the PSA response was 75.0% (95% CI, 47.6–92.7) with rezvilutamide 160 mg plus docetaxel and 60.0% (95% CI, 32.3–83.7) with rezvilutamide 240 mg plus docetaxel. PSA decrease ≥50% was noted in 27 (75.0%; 95% CI, 57.8–87.9) of the 36 evaluable patients who received one or more doses of the study treatment. PSA decrease ≥50% was 77.8% (95% CI, 52.4–93.6) with rezvilutamide 160 mg plus docetaxel and 72.2% (95% CI, 46.5–90.3) with rezvilutamide 240 mg plus docetaxel. Figure 1 presents the PSA reduction at the 12th week and the largest PSA decrease across both groups (Figure 1).

**TABLE 3:** Efficacy endpoints.

	Rezvilutamide 160 mg plus docetaxel (*n* = 18)	Rezvilutamide 240 mg plus docetaxel (*n* = 18)	Overall (*n* = 36)
PSA response			
No. of evaluable patients, *n*	16	15	31
PSA response at week 12, *n* (%; 95% CI)	12 (75.0; 47.6–92.7)	9 (60.0; 32.3–83.7)	21 (67.7; 48.6–83.3)
No. of evaluable patients, *n*	18	18	36
PSA decline ≥50% from baseline	14 (77.8; 52.4–93.6)	13 (72.2; 46.5–90.3)	27 (75.0; 57.8–87.9)
Radiological response			
No. of evaluable patients, *n*	4	9	13
Best overall response, *n* (%)			
Partial response	2 (50.0)	0	2 (15.4)
Stable disease	2 (50.0)	7 (77.8)	9 (69.2)
Progressive disease	0	1 (11.1)	1 (7.7)
Not evaluable	0	1 (11.1)	1 (7.7)
Objective response rate, *n* (%; 95% CI)	2 (50.0; 6.8–93.2)	0 (0; 0–33.6)	2 (15.4; 1.9–45.5)
Disease control rate, *n* (%; 95% CI)	4 (100.0; 39.8–100.0)	7 (77.8; 40.0–97.2)	11 (84.6; 54.6–98.1)
Time to PSA progression			
No. of evaluable patients, n	18	18	36
Events, *n* (%)	10 (55.6)	8 (44.4)	18 (50.0)
Median (95% CI), months	10.5 (5.6–11.3)	14.1 (4.1–21.4)	10.5 (5.6–14.1)
Radiological progression‐free survival			
No. of evaluable patients, *n*	18	18	36
Events, *n* (%)	9 (50.0)	11 (61.1)	20 (55.6)
Median (95% CI), months	13.8 (5.7–19.6)	8.5 (5.6–27.6)	13.8 (8.4–19.2)
Overall survival			
No. of evaluable patients, n	18	18	36
Events, *n* (%)	12 (66.7)	14 (77.8)	26 (72.2)
Median (95% CI), months	19.5 (12.9–27.4)	14.5 (9.9–19.9)	16.2 (12.9–22.5)

Abbreviations: CI, confidence interval; PSA, prostate‐specific antigen

**FIGURE 2:**
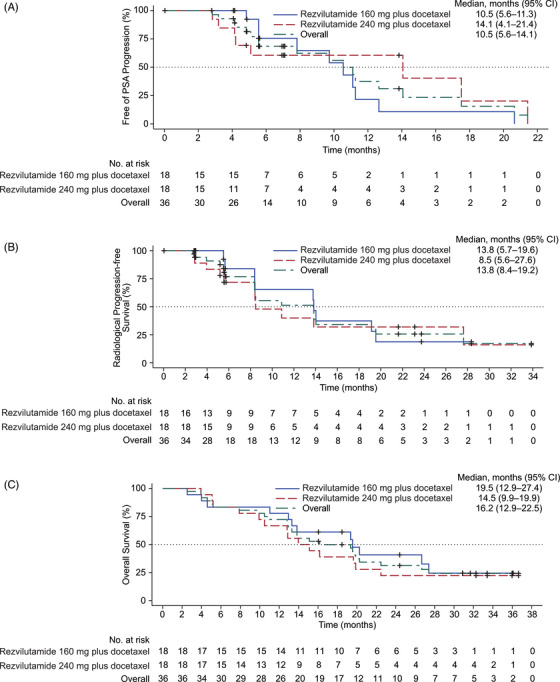
Kaplan‐Meier curves of time to PSA progression, radiological progression‐free survival and overall survival. (A) Time to PSA progression; (B) radiological progression‐free survival; (C) overall survival. CI, confidence interval; HR, hazard ratio; NR, not reached; PSA, prostate‐specific antigen.

By the data cutoff, 18 out of 36 evaluable patients (50.0%) experienced PSA progression (Table 3). Median time to PSA progression was 10.5 months (95% CI, 5.6–14.1) in overall population, with a duration of 10.5 months (95% CI, 5.6–11.3) for those receiving rezvilutamide 160 mg plus docetaxel, and 14.1 months (95% CI, 4.1–21.4) for those on rezvilutamide 240 mg plus docetaxel (Table 3; Figure 2A). Two (15.4%, 1.9–45.5) of the 13 evaluable patients who received one or more doses of the study treatment with measurable baseline target lesions achieved confirmed radiological response (Table 3). The ORR was 50.0% (95% CI, 6.8–93.2) with rezvilutamide 160 mg plus docetaxel and 0 (95% CI, 0–33.6) with rezvilutamide 240 mg plus docetaxel. The DCR was 84.6% (95% CI, 54.6–98.1) in all 13 evaluable patients, 100.0% (95% CI, 39.8–100.0) with rezvilutamide 160 mg plus docetaxel and 77.8% (95% CI, 40.0–97.2) with rezvilutamide 240 mg plus docetaxel. Figure S3 illustrates the optimal change in target lesions from baseline for both groups.

As of data cutoff, 20 (55.6%) of 36 evaluable patients experienced PFS events, including radiological progression or death (Table 3). The median radiological PFS for all patients was 13.8 months (95% CI, 8.4–19.2). Specifically, it was 13.8 months (95% CI, 5.7–19.6) with rezvilutamide 160 mg with docetaxel, and 8.5 months (95% CI, 5.6–27.6) with rezvilutamide 240 mg plus docetaxel (Table 3; Figure 2B). Twenty‐six (72.2%) patients died. The median OS for all patients was 16.2 months (95% CI, 12.9–22.5), 19.5 months (95% CI, 12.9–27.4) with rezvilutamide 160 mg plus docetaxel and 14.5 months (95% CI, 9.9–19.9) with rezvilutamide 240 mg plus docetaxel (Table 3; Figure 2C).

## 4 DISCUSSION

In this study, rezvilutamide plus docetaxel followed by maintenance with rezvilutamide monotherapy was safe for chemotherapy‐naive mCRPC patients who had failed abiraterone. What's more, rezvilutamide plus docetaxel followed by rezvilutamide maintenance led to a promising reduction in PSA, substantial tumour responses and improved survival.

The safety profile of rezvilutamide plus docetaxel in this study aligned with that of individual agents in mCRPC patients,^21^, ^24^ with no new safety signals observed. No one experienced DLT. The most frequently reported TRAEs were haematological toxicities, which might be mainly due to docetaxel. These events were manageable with standard supportive treatment and dose modification. Seizure is the most concerning safety issue associated with an AR antagonist.^25^, ^26^, ^27^ The study reported no instances of seizures of any grade. While the overall safety of rezvilutamide plus docetaxel was acceptable, the 240 mg rezvilutamide group had a higher incidence of grade ≥3 TRAEs, which may be related to the higher dose of rezvilutamide.

Studies involving docetaxel in chemotherapy‐naive mCRPC patients who had failed abiraterone showed a PSA decline of ≥50% in 13%–55% participants, with median times to PSA progression, radiological PFS, and OS being 4.6–5.6 months, 3.7–9.8 months and 12.4–16.9 months, respectively.^10^, ^11^, ^12^, ^13^, ^14^, ^15^ In our study, rezvilutamide plus docetaxel followed by rezvilutamide maintenance demonstrated a PSA response at week 12 of 67.7%, a rate of PSA decline ≥50% of 75.0%, a median time to PSA progression of 10.5 months, a median radiological PFS of 13.8 months, and a median OS of 16.2 months, which was higher than or similar to those previous studies of docetaxel in chemotherapy‐naive, abiraterone‐refractory, mCRPC patients. Of note, the median OS with rezvilutamide 160 mg plus docetaxel in our study was 19.5 months, which was superior to that observed in previous studies of docetaxel in this population. While cross‐trial comparisons require caution, rezvilutamide plus docetaxel followed by maintenance with rezvilutamide monotherapy shows therapeutic promise in chemotherapy‐naive mCRPC patients who had progressed after abiraterone.

A phase Ib clinical study of enzalutamide combined with docetaxel in mCRPC patients showed that all five patients who had failed previous abiraterone experienced PSA decline ≥50%.^20^ The rate of PSA decline ≥50% in our study (75.0%) aligns with the aforementioned study of novel AR antagonist plus docetaxel in similar patients. Our data further support rezvilutamide plus docetaxel followed by maintenance with rezvilutamide monotherapy for chemotherapy‐naive mCRPC patients who had progressed after abiraterone.

Taxanes are metabolised by the CYP3A4 enzyme.^28^ Rezvilutamide is a strong inducer of CYP3A.^21^ In this study, the geometric mean cycle 2/cycle 1 ratios of AUC_0–24 h_ and C_max_ for docetaxel were .735 and .781 with 160 mg of rezvilutamide, and .591 and .680 with 240 mg of rezvilutamide, indicating a stronger impact of the CYP3A strong inducer rezvilutamide on CYP3A4 substrate docetaxel at the higher dose. The reduced docetaxel exposure in the rezvilutamide 240 mg group may explain the efficacy disadvantage observed relative to the 160 mg group.

This study is subject to several limitations. The small sample size limited the precision of efficacy endpoint estimates. The study was also limited by its non‐randomised design and the absence of a control group. Part 2 of this study was designed as a randomised, controlled, open‐label, multi‐centre clinical trial, but it was discontinued due to a strategic portfolio reprioritisation for rezvilutamide. Consequently, the development strategy has been redirected toward investigating its synergistic potential in combination with antibody‐drug conjugates (ADCs). For example, a recently initiated randomised phase II clinical study is now evaluating rezvilutamide plus HS‐20093, a B7‐H3‐targeting ADC, in metastatic prostate cancer (NCT07230106).

## 5 CONCLUSIONS

Rezvilutamide plus docetaxel followed by maintenance with rezvilutamide monotherapy was safe and effective in chemotherapy‐naive mCRPC patients who had progressed after abiraterone. Rezvilutamide at 240 mg has a stronger influence on docetaxel than rezvilutamide at 160 mg. These results support the further investigation of rezvilutamide plus docetaxel versus docetaxel alone for chemotherapy‐naive mCRPC patients who had progressed after abiraterone.

## AUTHOR CONTRIBUTIONS

Full access to all the data in the study and responsibility for the integrity of the data and the accuracy of the data analysis: Zhenhua Liu, Bo Chen, Ping Feng and Qiang Wei. Study concept and design: Qiang Wei. Acquisition of data: Zhenhua Liu, Bo Chen, Tao Dai, Shusuan Jiang, Hong Luo, Hong Liao, Dexin Yu, Zhiwen Chen, Dahong Zhang, Zeng Li, Zhiqiang Zhang, Hong Bai, Ping Feng and Qiang Wei. Analysis and interpretation of data: All authors. Drafting of the manuscript: Zhenhua Liu, Bo Chen, Feng Liu, Jiaqin Lin and Yike Wang. Critical revision of the manuscript for important intellectual content: All authors. Statistical analysis: Jiaqin Lin. Obtaining funding: Qiang Wei. Administrative, technical or material support: Zhenhua Liu, Bo Chen, Tao Dai, Shusuan Jiang, Hong Luo, Hong Liao, Dexin Yu, Zhiwen Chen, Dahong Zhang, Zeng Li, Zhiqiang Zhang, Hong Bai, Feng Liu, Jiaqin Lin, Yike Wang and Ping Feng. Supervision: Qiang Wei. Other: None.

## CONFLICT OF INTEREST STATEMENT

Feng Liu, Jiaqin Lin and Yike Wang reported being employed by Jiangsu Hengrui Pharmaceuticals Co., Ltd. No other disclosures were reported.

## ETHICS STATEMENT

The study was conducted in accordance with the Declaration of Helsinki and Good Clinical Practice guidelines. The protocol and all amendments were approved by the Ethics Committee of West China Hospital (2020‐133), Hunan Cancer Hospital (2021‐562), Chongqing University Cancer Hospital (CZLS2020252‐D), Sichuan Cancer Hospital (GCP‐XY‐2021‐001‐01), Second Affiliated Hospital of Anhui Medical University (YJ‐YW2020‐157‐2), the First Affiliated Hospital of the Chinese People's Liberation Army Medical University ([BA]YW202284) and Zhejiang Provincial People's Hospital (2020YW121‐A2).

## Supporting information



Supporting information

## Data Availability

Data are available from the corresponding authors upon reasonable request.
