# Supplementary material for: Rezvilutamide plus docetaxel in chemotherapy‐naive metastatic castration‐resistant prostate cancer patients after progression on abiraterone: A multi‐centre, open‐label, phase II trial
Source: Clin Transl Med. 2026 Apr 16;16(4):e70649. doi: 10.1002/ctm2.70649 (PMC13084187; doi:10.1002/ctm2.70649)
Supplement: Supplementary file 1 — Supporting information [file CTM2-16-e70649-s001.docx]

# Appendix

## Table of contents

[Appendix 1](#_Toc224048391)

[Table of contents 1](#_Toc224048392)

[Table S1. Summary of the subsequent systemic antitumor treatment 2](#_Toc224048393)

[Table S2. Summary of adverse events 3](#_Toc224048394)

[Table S3. Treatment-related serious adverse events 5](#_Toc224048395)

[Table S4. Adverse events leading to death 6](#_Toc224048396)

[Table S5. Pharmacokinetic parameters of docetaxel, when combined with rezvilutamide 8](#_Toc224048397)

[Figure S1. Patient disposition 9](#_Toc224048398)

[Figure S2. Mean plasma concentration versus time curve of docetaxel, when combined with rezvilutamide 10](#_Toc224048399)

[Figure S3. The best percentage change from baseline in target lesions 11](#_Toc224048400)

[Supplemental methods 12](#_Toc224048401)

[Procedures 12](#_Toc224048402)

[Outcomes 12](#_Toc224048403)

[Assessments 13](#_Toc224048404)

[Statistical analysis 13](#_Toc224048405)

## Table S1. Summary of the subsequent systemic antitumor treatment

|  | **Rezvilutamide 160 mg plus docetaxel**  **(n=18)** | **Rezvilutamide 240 mg plus docetaxel**  **(n=18)** | **Overall**  **(n=36)** |
| --- | --- | --- | --- |
| Subsequent antitumor therapy (except ADT) | 6 (33.3) | 4 (22.2) | 10 (27.8) |
| Abiraterone | 1 (5.6) | 0 | 1 (2.8) |
| Docetaxel | 1 (5.6) | 0 | 1 (2.8) |
| Novel AR inhibitor | 1 (5.6) | 1 (5.6) | 2 (5.6) |
| Other | 3 (16.7) | 3 (16.7) | 6 (16.7) |

Data are n (%). ADT, androgen deprivation therapy; AR, androgen receptor.

## Table S2. Summary of adverse events

|  | **Rezvilutamide 160 mg**  **plus docetaxel**  **(n=18)** | **Rezvilutamide 240 mg**  **plus docetaxel**  **(n=18)** | **Overall**  **(n=36)** |
| --- | --- | --- | --- |
| AEs |  |  |  |
| Treatment-emergent | 18 (100.0) | 18 (100.0) | 36 (100.0) |
| Treatment-related | 18 (100.0) | 18 (100.0) | 36 (100.0) |
| Serious AEs |  |  |  |
| Treatment-emergent | 9 (50.0) | 10 (55.6) | 19 (52.8) |
| Treatment-related | 7 (38.9) | 5 (27.8) | 12 (33.3) |
| Grade ≥3 AEs |  |  |  |
| Treatment-emergent | 16 (88.9) | 18 (100.0) | 34 (94.4) |
| Treatment-related | 14 (77.8) | 18 (100.0) | 32 (88.9) |
| AEs leading to treatment discontinuation |  |  |  |
| Treatment-emergent | 1 (5.6) | 8 (44.4) | 9 (25.0) |
| Treatment-related | 1 (5.6) | 5 (27.8) | 6 (16.7) |
| AEs leading to treatment discontinuation of rezvilutamide |  |  |  |
| Treatment-emergent | 0 | 2 (11.1) | 2 (5.6) |
| Treatment-related | 0 | 0 | 0 |
| AEs leading to treatment discontinuation of docetaxel |  |  |  |
| Treatment-emergent | 1 (5.6) | 6 (33.3) | 7 (19.4) |
| Treatment-related | 1 (5.6) | 5 (27.8) | 6 (16.7) |
| AEs leading to treatment discontinuation of prednisone |  |  |  |
| Treatment-emergent | 1 (5.6) | 4 (22.2) | 5 (13.9) |
| Treatment-related | 1 (5.6) | 3 (16.7) | 4 (11.1) |
| AEs leading to dose reduction or treatment interruption |  |  |  |
| Treatment-emergent | 6 (33.3) | 9 (50.0) | 15 (41.7) |
| Treatment-related | 6 (33.3) | 7 (38.9) | 13 (36.1) |
| AEs leading to dose reduction or treatment interruption of rezvilutamide |  |  |  |
| Treatment-emergent | 4 (22.2) | 6 (33.3) | 10 (27.8) |
| Treatment-related | 4 (22.2) | 3 (16.7) | 7 (19.4) |
| AEs leading to dose reduction or treatment interruption of docetaxel |  |  |  |
| Treatment-emergent | 4 (22.2) | 6 (33.3) | 10 (27.8) |
| Treatment-related | 3 (16.7) | 6 (33.3) | 9 (25.0) |
| AEs leading to dose reduction or treatment interruption of prednisone |  |  |  |
| Treatment-emergent | 3 (16.7) | 3 (16.7) | 6 (16.7) |
| Treatment-related | 1 (5.6) | 3 (16.7) | 4 (11.1) |
| AEs leading to death |  |  |  |
| Treatment-emergent | 2 (11.1) | 1 (5.6) | 3 (8.3) |
| Treatment-related | 0 | 0 | 0 |

Data are n (%). AE, adverse event.

## Table S3. Treatment-related serious adverse events

|  | **Rezvilutamide 160 mg**  **plus docetaxel**  **(n=18)** | **Rezvilutamide 240 mg**  **plus docetaxel**  **(n=18)** | **Overall**  **(n=36)** |
| --- | --- | --- | --- |
| Any | 7 (38.9) | 5 (27.8) | 12 (33.3) |
| Pneumonia | 1 (5.6) | 2 (11.1) | 3 (8.3) |
| Anaemia | 2 (11.1) | 0 | 2 (5.6) |
| Decreased appetite | 1 (5.6) | 1 (5.6) | 2 (5.6) |
| Blood infection | 1 (5.6) | 0 | 1 (2.8) |
| Coronary artery disease | 1 (5.6) | 0 | 1 (2.8) |
| Febrile neutropenia | 0 | 1 (5.6) | 1 (2.8) |
| Hematuria | 0 | 1 (5.6) | 1 (2.8) |
| Interstitial lung disease | 0 | 1 (5.6) | 1 (2.8) |
| Neutropenia | 1 (5.6) | 0 | 1 (2.8) |
| Pneumonia bacterial | 1 (5.6) | 0 | 1 (2.8) |
| Sepsis | 1 (5.6) | 0 | 1 (2.8) |
| Urinary tract infection | 1 (5.6) | 0 | 1 (2.8) |
| White blood cell count decreased | 0 | 1 (5.6) | 1 (2.8) |

Data are n (%).

## Table S4. Adverse events leading to death

|  | **Respiratory-circulatory failure** | **Tumor progression** | **Tumor progression** |
| --- | --- | --- | --- |
| **Patient ID** | 01002 | 01020 | 18004 |
| **Patient baseline** | A 74-year-old male patient with mCRPC complicated by hyperlipidemia. | A 75-year-old male patient with mCRPC, presenting with baseline pulmonary metastasis, multiple systemic bone metastases, complicated by type 2 diabetes mellitus and bone pain. | A 58-year-old male patient with mCRPC. |
| **Date of treatment start** | Dec. 17, 2020 | Jun. 29, 2021 | Feb. 25, 2021 |
| **Date of rezvilutamide discontinuation** | Jul. 25, 2022 | Sep. 28, 2021 | Early June 2021 |
| **Date of docetaxel discontinuation** | Jun. 24, 2021 | Aug. 10, 2021 | May. 19, 2021 |
| **Date of prednisone discontinuation** | Sep. 4, 2021 | Aug. 10, 2021 | May. 19, 2021 |
| **Date of death** | Aug. 3, 2022 | Oct. 28, 2021 | Jun. 24, 2021 |
| **Is the AE related to the treatment? Why?** | No. An elderly mCRPC patient, with the last tumor efficacy assessment showing disease progression, and the likelihood of death considered largely attributable to disease progression, unlikely related to rezvilutamide. Since both docetaxel and prednisone were discontinued in 2021, there is no temporal association with the occurrence of this AE, and it is considered definitely unrelated to docetaxel and prednisone. | No. The patient died from multiorgan failure due to worsening metastatic disease. The death attributable to the natural progression of metastatic prostate cancer, not to drug-related toxicity. This AE is unlikely related to rezvilutamide, docetaxel, and prednisone. | No. Death occurred after discontinuation of all study drugs, with symptoms related to disease progression (generalized fatigue, anorexia, abdominal distension, and shortness of breath). The event was consistent with end-stage disease progression. This AE is unlikely related to rezvilutamide, docetaxel, and prednisone. |

AE, adverse event; mCRPC, metastatic castration-resistant prostate cancer.

## Table S5. Pharmacokinetic parameters of docetaxel, when combined with rezvilutamide

|  | **Rezvilutamide 160 mg plus docetaxel** | | **Rezvilutamide 240 mg plus docetaxel** | |
| --- | --- | --- | --- | --- |
|  | **n** | **value** | **n** | **value** |
| Cycle 1 |  |  |  |  |
| AUC_0-24h_ (h*ng/mL) | 17 | 2310 (50.0) | 17 | 2320 (67.8) |
| C_max_ (ng/mL) | 17 | 2030 (49.7) | 17 | 1990 (54.5) |
| C_24h_ (ng/mL) | 15 | 8.29 (51.8) | 16 | 9.55 (58.6) |
| Cycle 2 |  |  |  |  |
| AUC_0-24h_ (h*ng/mL) | 17 | 1700 (32.2) | 16 | 1360 (65.8) |
| C_max_ (ng/mL) | 17 | 1590 (26.8) | 16 | 1340 (70.7) |
| C_24h_ (ng/mL) | 11 | 5.86 (29.0) | 10 | 6.16 (14.4) |
| Cycle 2/cycle 1 ratio |  |  |  |  |
| AUC_0-24h_ | 17 | 0.735 (47.3) | 16 | 0.591 (74.4) |
| C_max_ | 17 | 0.781 (50.3) | 16 | 0.680 (69.5) |
| C_24h_ | 10 | 0.626 (41.0) | 9 | 0.482 (56.8) |

Data are Geometric mean (GeoCV%). Cycle 2/cycle 1, cycle 2 versus cycle 1; GeoCV%, percentage geometric coefficient of variation.

## Figure S1. Patient disposition


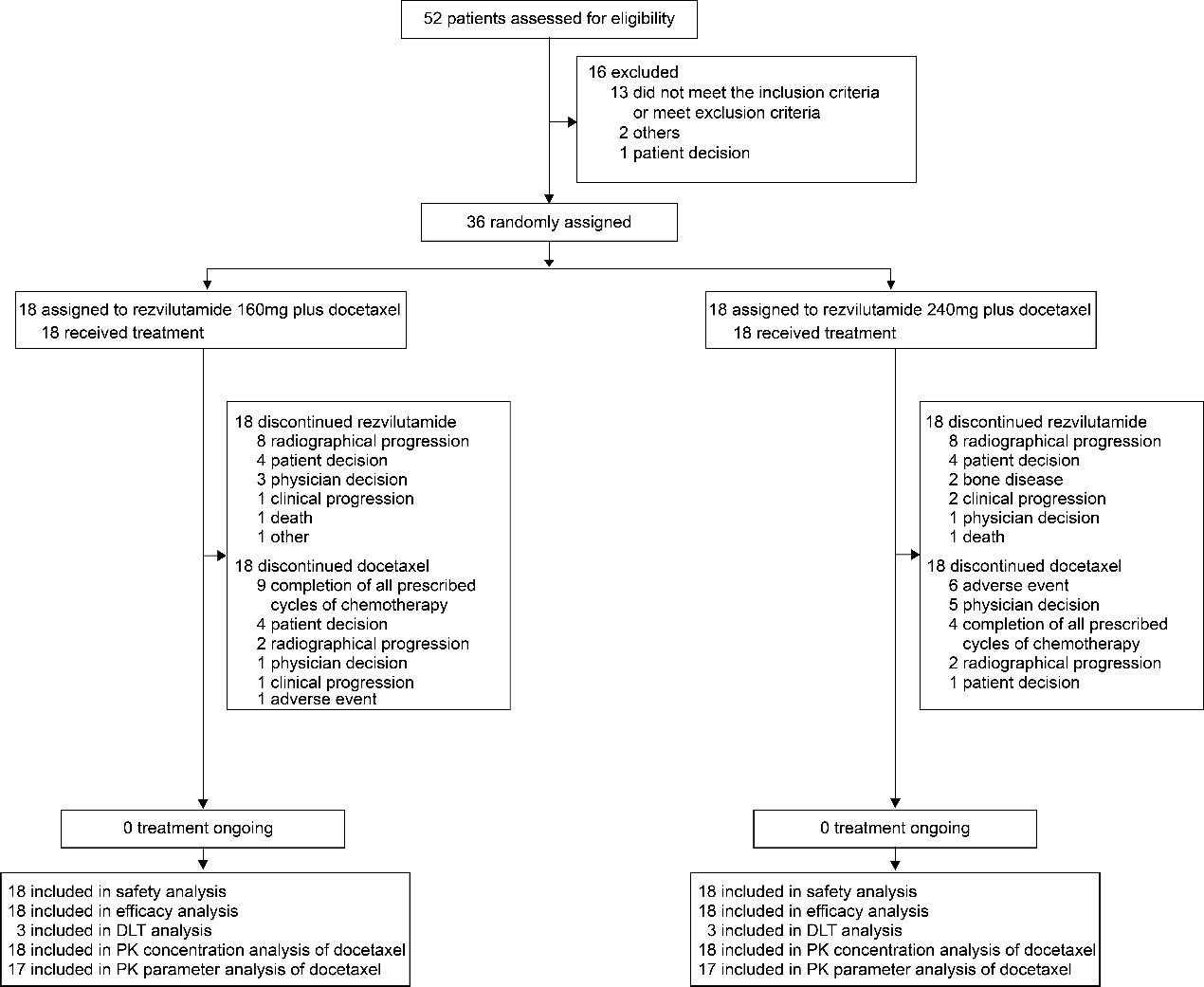


DLT, dose-limiting toxicity; PK, pharmacokinetics.

## Figure S2. Mean plasma concentration versus time curve of docetaxel, when combined with rezvilutamide


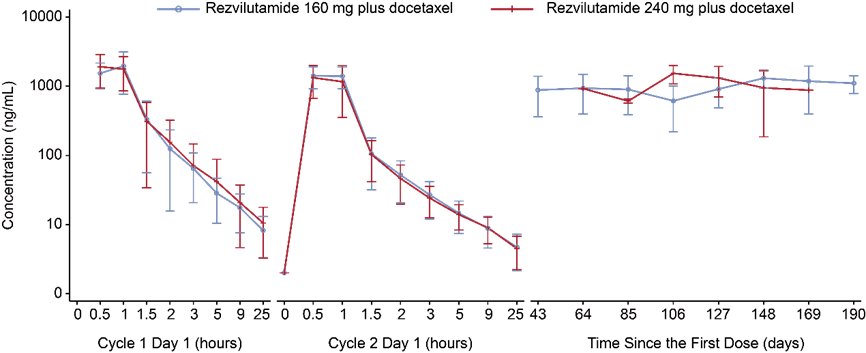


## Figure S3. The best percentage change from baseline in target lesions


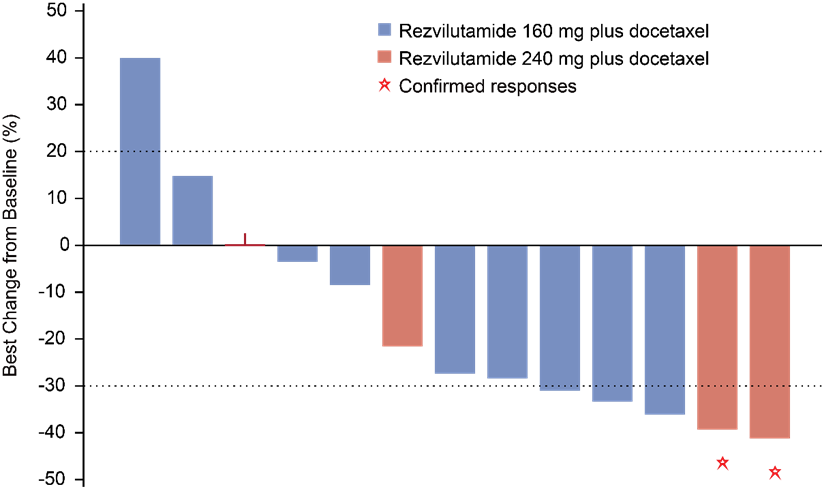


## Supplemental methods

### Procedures

Treatment interruptions and dose reduction of rezvilutamide and docetaxel were allowed to manage toxicities. For rezvilutamide, each interruption should not exceed 14 days. For docetaxel, resumption of treatment within 7 days after interruption was recommended; if the interruption lasted for more than 21 days, the investigator would judge, based on the benefit to the patient, whether the patient should continue with the chemotherapy. The dose of prednisone could remain unchanged when the doses of rezvilutamide and docetaxel are adjusted; if docetaxel is discontinued, prednisone should also be discontinued.

### Outcomes

PSA response at week 12 was defined as a decrease of ≥50% in PSA at week 12 from baseline. Objective response rate was defined as the proportion of patients who achieved a confirmed complete response or partial response as the best overall response for soft tissue lesions (per RECIST version 1.1) and showed no progression of bone disease (per PCWG3 criteria). Disease control rate was defined as the proportion of patients who achieved a confirmed complete response, confirmed partial response, or stable disease as best overall response based on RECIST version 1.1. Time to PSA progression was the time from the start of study treatment to the first observation of PSA progression, excluding changes in PSA levels within the first 12 weeks of treatment. If PSA level at week 12 is below the baseline level, PSA progression is defined as a ≥25% increase and an absolute increase of ≥2 ng/L above the lowest post-week 12 level. If PSA level at week 12 is not below the baseline level, PSA progression is defined as a ≥25% increase and an absolute increase of ≥2 ng/L above the baseline level. PSA progression must be confirmed at least 3 weeks later. Radiological progression-free survival was the time from the start of study treatment to the first radiographic progression or death. Radiographic progression includes soft tissue progression (per RECIST version 1.1) and bone progression (per PCWG3 criteria). Overall survival was the time from the initiation of study treatment to death.

### Assessments

DLTs were defined as any of the following treatment-related adverse events (TRAEs): grade ≥3 non-hematological toxicity (excluding grade ≥3 laboratory abnormalities that do not require hospitalization and grade ≥3 rash, nausea, vomiting, or diarrhea that persisted for ˂3 days with symptomatic treatment); grade 3 decreased platelet count that persisted for ≥7 days; grade 3 decreased platelet count with bleeding; grade 4 decreased platelet count; grade ≥3 febrile neutropenia (body temperature >38.3 ℃); grade 4 decreased neutrophil count that persisted for >5 days despite symptomatic treatment; and grade 4 anaemia.

Blood samples for PK analysis of docetaxel were collected before the infusion of docetaxel, at 0.5 hours during infusion, within 5 minutes, 0.5, 1, 2, 4, 8, 24 hours after the end of infusion during the first and second cycles, and within 5 minutes after the end of infusion during cycle 3-10, with 3 mL of venous blood collected at each time point. Blood samples for PK analysis of rezvilutamide were collected 30 minutes before the administration of rezvilutamide in the first day of cycle 2-4, with 2 mL of venous blood collected at each time point.

### Statistical analysis

DLTs were assessed in all patients who enrolled in the dose-escalation phase and completed the 21-day evaluation period or experienced any DLT during the period. Safety and efficacy (except PSA response at week 12, ORR, and DCR) were analysed in patients who received at least one dose of the study treatment. PSA response at week 12 was analysed in patients who received at least one dose of the study treatment and had at least one PSA level measurement at week 12 and later. ORR and DCR were analysed in patients who received at least one dose of the study treatment and had measurable target lesions at baseline. PK concentration and PK parameter analyses were performed in patients who received at least one dose of the study treatment and had at least one qualified PK concentration and PK parameter result.

The point estimates of PSA response at week 12, rate of PSA decline ≥50% from baseline, objective response rate, and disease control rate were provided as well as their two-sided 95% CIs calculated using the Clopper-Pearson method. Time-to-event outcomes including time to PSA progression, radiological progression-free survival, and overall survival were estimated using the Kaplan-Meier method, and their two-sided 95% CIs were calculated with the Brookmeyer-Crowley method.
